# Bioassay-guided isolation of active anti-adipogenic compound from royal jelly and the study of possible mechanisms

**DOI:** 10.1186/s12906-018-2423-2

**Published:** 2019-01-29

**Authors:** Prakash Raj Pandeya, Ramakanta Lamichhane, Kyung-Hee Lee, Se-Gun Kim, Dae-Ho Lee, Hyeong-Kyu Lee, Hyun-Ju Jung

**Affiliations:** 1 Department of Oriental Pharmacy and Wonkwang-Oriental Medicines Research Institute, Wonkwang University, Sinyong-Dong, Iksan, 570-749 South Korea; 2 Department of Agricultural Biology, National Institute of Agricultural Science, Rural Development Administration, Wanju, 55365 South Korea; 3 Department of Internal Medicine, Gachon University Gil Medical Centre, Incheon, 21565 South Korea; 4 Natural Medicine Research Center, Korea Research Institute of Bioscience and Biotechnology, Yeongudanji-ro 30, Ochang-eup, Cheongju-si, 28116 South Korea; 5 College of Pharmacy, Wonkwang University, PO. Box – 54538, 460, Iksan-daero, Iksan-si, Jeonbuk Republic of Korea

**Keywords:** Royal jelly, (*E*)-10- Hydroxy-2-decenoic acid, 3 T3-L1 adipocyte, cAMP/PKA pathway, Insulin signaling pathway

## Abstract

**Background:**

Royal jelly (RJ) has been used traditionally for dietary, cosmetic and health purposes for a long time in different parts of the world. Scientific studies have also shown its numerous health-promoting properties including hypoglycemic and anti-hypercholesterolemic action. In this study, we investigated the anti-adipogenic activity of RJ in 3 T3-L1 cells and isolated the major responsible root component for the activity.

**Methods:**

An active anti-adipogenic compound was isolated through bioassay-guided isolation process by successive treatment of RJ and its active fractions on 3 T3-L1 cell line. (*E*)-10-Hydroxy-2-decenoic Acid (10-HDA) was identified using NMR spectroscopy and ultra-performance liquid chromatography (UPLC). As 10-HDA showed significant anti-adipogenic activity with Oil Red O staining and TG content assay on 3 T3-L1 adipocytes, further study was carried out in molecular level for the expression of adipogenic transcription factors such as PPARγ, FABP4, C/EBPα, SREBP-1c, and Leptin. The effect of 10-HDA on preliminary molecules such as pAkt, pERK, C/EBPβ, and pCREB were studied in the early stage of adipogenesis. The effect of 10-HDA on reactive oxygen species (ROS) production in fully differentiating adipocytes was measured by nitro blue tetrazolium (NBT) assay.

**Result:**

Results showed that triacylglycerol accumulation and ROS production was markedly suppressed by 10-HDA. Preliminary molecules such as pAkt, pERK, pCERB, and C/EBPβ were found to be down-regulated by 10-HDA, which led to down-regulation of key adipogenic transcription factors such as PPARγ, FABP4, CEBPα, SREBP-1c, and Leptin on 3 T3-L1 adipocytes.

**Conclusion:**

Our results suggest that anti-adipogenesis of 10-HDA on 3 T3-L1 adipocyte takes place via two mechanisms: inhibition of cAMP/PKA pathway and inhibition of p-Akt and MAPK dependent insulin signaling pathway. So it is considered that 10-HDA, a major component of RJ, can be a potential therapeutic medicine for obesity.

**Electronic supplementary material:**

The online version of this article (10.1186/s12906-018-2423-2) contains supplementary material, which is available to authorized users.

## Background

Royal jelly (RJ) is a hypopharyngeal and mandibular secretion of the worker honeybee *Apis mellifera* L., which is mainly secreted between the sixth and twelfth days of their life [1]. RJ has been associated to be involved in the longer lifespan of queens in contrast with workers honeybee [2]. RJ is considered a source of multiple nutrients. High nutritional value of RJ is due to the unique composition of all five nutritive and body-building materials (proteins, fats, carbohydrates, vitamins and minerals) along with roalactin, the main inducer protein, which allows larva to morph into long-lived queen bee [2, 3]. RJ is one of the most attractive ingredients for the preparation of healthy functional foods, due to being a packet of several health promoting properties such as growth promoting, antiaging, hypoglycaemic, anti-hypercholesterolemic, radio-protective, gastro-protective, hepato-protective, antitumor, anti-inflammatory, vasodilative and hypotensive, antioxidant, antimicrobial, and disinfectant action [4]. Thus, RJ is widely utilized in food and pharmaceutical industries nowadays.

Though limited scientific publications are available, RJ has been traditionally used for dietary, cosmetic and health purposes from a long time in different parts of the world. In China and Japan, it has been used as medicine to keep blood sugar level normal. It is believed that RJ can prolong life, a similar effect as in queen bee. Also, it has been traditionally known to increase energy, reduce hypertension, improve memory, reduce anxiety and prevent senility [5]. RJ has been used for a long time to improve menopausal symptoms [6, 7].

The composition of freeze-dried RJ showed 8–19% of lipid. A major portion of the lipid composed of (*E*)-10-Hydroxy-2-decenoic acid (10-HDA), which is more than 3.5% of freeze-dried RJ [8, 9]. Butenandt separated 10-HDA from RJ in 1956 for the first time [10], and later in 1959, Barker determined its chemical constitution and configuration by infrared spectrum and NMR [11]. Now, 10-HDA is considered as a most important compound in quality evaluation of RJ in the international standard [3]. According to Chinese quality standards, RJ should contain not less than 1.4% of 10- HDA as a quality product [12].

Obesity is the expansion of unnecessary mass in white adipose tissue (WAT) [13]. It is a major causative factor for multiple metabolic disorders such as insulin resistance (type2 diabetes), dyslipidemia and hypertension. Other diseases such as heart failure, fatty liver disease, obstructive sleep apnea, coronary artery disease, atherosclerosis, gallstones, stroke, reproductive, and gastrointestinal cancers are also the consequences of obesity [14]. Obesity and extreme weight gain are mentioned in the fifth leading risk factors for global mortality [15]. Obesity results from both increased size (hypertrophy) and number (hyperplasia) of the adipocytes in adipose tissue [16]. Hypertrophy of adipocytes occurs by accumulation of excess triacylglycerol (TG) and free fatty acids (FFA). And hyperplasia results from the over differentiation of preadipocytes into adipocytes [13, 17]. Preadipocytes are differentiated through the process of adipogenesis [18]. The differentiated adipose cells in WAT exhibit various morphological and biochemical physiognomies such as accumulation of TG, regulation of insulin metabolism and expression of adipogenic genes, such as CAAT/enhancer binding protein alpha (C/EBPα), peroxisome proliferator-activated receptor gamma (PPARγ), fatty acid synthase (FAS), lipoprotein lipase (LPL), sterol response element binding protein- 1c (SREBP-1c) and adipocyte-specific fatty acid binding proteins (FABP4) [19, 20]. PPARγ is a principal transcription factor that stimulates and maintains the expression of several other genes essential for adipogenesis [21].

There are many reports on pharmacological activities of RJ in the experimental cell line, but the anti-adipogenic property has not been reported yet. The aim of this study was to investigate the anti-adipogenic root of RJ in the 3 T3-L1 cell line and to find out the possible mechanisms of the most active compound.

## Methods

### Materials and reagents

RJ was obtained from Yeoju Honey Park, Korea and was stored at − 20 °C until use. ^1^H-NMR and ^13^C NMR of the isolated compound were recorded on JEOL Eclipse 500 FT-NMR spectrometer (^1^H-NMR, 500 MHz) using a deuterated solvent (DMSO-*d*_6_ containing 0.03% TMS). High-resolution electrospray ionization mass spectrometry (HR-ESIMS) were performed on Water Q-Tof Premnier Mass Spectrometer (Micromass UK Ltd., Manchester, UK). The purity of the isolated compound was assessed using ultra-performance liquid chromatography (UPLC) system (Agilent Technologies, Germany) equipped with a pump (1290 Bin Pump), auto-sampler, and photodiode detector (1290 DAD). Standard (E)-10-Hydroxy-2-decenoic Acid (10-HDA) was purchased from Nacalai Tesque, Inc. (Kyoto, Japan). Mouse embryonic fibroblasts, adipose like cell line (3 T3-L1 preadipocytes) were purchased from American Type Culture Collection (ATCC). Dulbecco's Modified Eagle Medium (DMEM), newborn calf serum (NCS) and fetal bovine serum (FBS) were purchased from Gibco, USA. 3-Isobutyl-1-methylxanthine (IBMX), dexamethasone, insulin, 10% formalin, isopropanol and Oil-red O (ORO) were purchased from Sigma-Aldrich (St. Louis, MO, USA). The Cleantech TG-S (Triglyceride) kit for TG assay was obtained from Asan Pharmaceutical, South Korea. The thiazolyl blue tetrazolium bromide (MTT) was purchased from Alfa Aesar, England. The dimethyl sulfoxide (DMSO) was purchased from Junsei, Japan. The Qiazol lysis reagent for RNA extraction was purchased from Qiagen Sciences (Maryland, USA). High capacity RNA-to-cDNA kit and power SYBR-Green PCR master mix were purchased from Applied Biosystems, UK. The protein extraction solution was purchased from iNtron Biotechnology, Korea. Anti-rabbit (Millipore, USA) and anti-mouse (thermos scientific, USA) were used as secondary horseradish peroxidase-conjugated antibody. Primary antibodies against PPARγ, A-FABP (B-4), C/EBPα, C/EBPβ, and GAPDH were purchased from Santa Cruz Biotechnology, Inc. Rest of the primary antibodies against phospho-Akt, Akt, phospho-ERK1/2, ERK1/2, phosphor-CERB, and CREB were purchased from Cell Signaling Technology.

### Isolation of anti-adipogenic compound from RJ

The bioassay-guided isolation was initiated from the assessment of the anti-adipogenic activity of RJ. As RJ showed significant anti-adipogenic activity on 3 T3-L1 adipocytes, further isolation of the active compound was directed. For this study 95.5 g of RJ having water content 67.58% was suspended into the water in the ratio of 1: 50 (*w*/*v*), and subjected to fractionation using ethyl acetate (EA) to obtain EA and water fractions. Both of the fractions were tested in 3 T3-L1 adipocytes. As EA fraction showed significant anti-adipogenic activity, isolation of a major compound (from TLC pattern) from EA fraction was performed using normal phase chromatography followed by reverse phase chromatography. EA fraction (4.96 g) was wet loaded on a normal phase Silica gel column (35.5 × 3.5 cm) by dissolving on a small amount of eluting solvent mixture (dichloromethane: methanol: water = 9:1.5:1, *v*/v). The sub-fractions having similar TLC pattern were mixed and dried to give five fractions (Fr. 1–5). Fraction 3 (3.49 g) was subjected to reverse phase column chromatography for further isolation of the major compound. The column was eluted with 50% acetonitrile with water. The Eluents having similar TLC patterns were collected and concentrated. The major compound (715.45 mg) was obtained in pure form. The compound was tested in the 3 T3-L1 cells. As the compound showed potential anti-adipogenic activity, the structure of the compound was identified by using chromatographic and nuclear magnetic resonance (NMR) techniques. The ^1^H NMR and ^13^C NMR of the isolated compound were recorded using a deuterated solvent (DMSO-*d*_6_ containing 0.03% TMS). The molecular mass of the compound was assessed using HR-ESIMS. The purity of the isolated compound was assessed using thin layer chromatography (TLC) and ultra-performance liquid chromatography (UPLC) by comparing with standard 10-HDA.

### Sample preparation for cell treatment

Ethyl acetate (EA) fraction of RJ and 10-HDA stock samples in 50 mg/mL concentration were prepared by dissolving in DMSO; whereas freeze-dried RJ was suspended into 50% DMSO/PBS to make 50 mg/mL stock concentration. Stock samples were preserved at − 20 °C until use. Samples in required concentration were prepared by diluting stock sample into respective media immediately before the cell treatment. The DMSO concentration on media treated with 10-HDA, RJ-EA fraction and RJ was below 0.4, 0.6, and 0.1% respectively.

### Cell culture and measurement of cell toxicity

3 T3-L1 preadipocyte cell culture and sample toxicity ware performed as described by Lamichhane et al., 2017 [22]. Cells were grown and passaged in DMEM containing 10% NCS and 1% penicillin/streptomycin/amphotericin B in a humidified atmosphere of 5% CO_2_ at 37 °C. The 3 T3-L1 preadipocytes were seeded into 48 well plates at a density of 1.2 × 10^6^ cells /mL (6 × 10^5^ cells/ well) in DMEM supplemented with 10% NCS. The cells were treated with various concentrations of RJ, EA fraction, water fraction and 10-HDA compound for 48 h. The sample toxicity to the cell was evaluated by the MTT assay.

### Adipocyte differentiation and ORO staining

Adipocyte differentiation and ORO staining were done according to the method described by Lamichhane et al., 2017 with slight modification [22]. The 3 T3-L1 preadipocytes were seeded into 6 well plates at a density of 2 × 10^5^ cells /ml (6 × 10^5^ cells/ well) in DMEM supplemented with 10% NCS and incubated at 37 °C in a 5% CO_2_ incubator. The media was replaced in every alternate day until the cells become confluence. After 2 days of the confluence (Day 0), differentiation of the 3 T3-L1 preadipocytes was induced by differentiation medium (MDI) containing 0.5 mM IBMX, 1 μM Dexamethasone and 5 μg/mL insulin in DMEM containing 10% FBS. Each cell group was treated with MDI media including respective sample at Day 0 and incubated for 48 h. The 10-HDA, RJ-EA fraction and RJ were treated below the concentration of 200 μg/mL, 300 μg/mL and 1 mg/mL respectively. Therefore the final concentration of DMSO on cell culture was below 0.4, 0.6, and 1% respectively. Adipocyte maintenance medium containing 5 μg/mL insulin and 10% FBS in DMEM was replaced for two times in every two-day interval (Day 2 and Day 4). At Day 6 of post-treatment previous media was replaced with DMEM with 10% FBS and maintained for the next 2 days. Then the cellular lipid content was assessed by Oil-red O (ORO) staining.

### Triacylglycerol (TG) content assay

The intracellular TG was assessed at Day 8 of differentiation. After two washes with ice-cold PBS, the cells were collected using 0.1 mL of cell lysis buffer. The collected cells were then vertex for 5 min and centrifugation at 15,000 g for 15 min at 4 °C. The supernatants were assayed for triglyceride content according to the manufacturer’s protocol [TG-S (Triglyceride) kit] with some modifications. TG assay buffer (180 μL) and cell lysate (50 μL) was mixed and incubated for 1 h at 37 °C and then absorbance was measured at 540 nm wavelength using a microplate reader.

### Protein extraction and western blotting

Western blotting was performed as described by Lee et al., 2017 [23]. Cell lysates were harvested in the different time frame during the differentiation period for western blot analysis of various proteins. Cells in six-well plates were washed with ice-cold PBS and exposed immediately into ice-cold protein extraction solution. Lysates were intermittently vertex for 10 min and centrifuged at 16000×g for 15 min at 4 °C. Total protein concentration was quantified according to the method of Bradford, using BSA (bovine serum albumin) as a standard. Sample proteins were denatured for 5 min at 95 °C and subjected to SDS/PAGE gel and transferred to nitrocellulose membrane. The membranes were blocked in 5% non-fat milk in TBS 0.02% Tween 20 (TBS-T) for 1 h at room temperature. After washing with TBS-T, the membrane was incubated at 4 °C with specific primary antibodies for 12 h. Next, the membranes were washed extensively with TBS-T and incubated with the secondary anti-rabbit/mouse horseradish peroxidase-conjugated antibody for 1 h at room temperature. Bands were visualized by enhanced chemiluminescence (ECL) solution. Primary antibodies against PPARγ, FABP4, C/EBPα, C/EBPβ, GAPDH, phospho-Akt, total-Akt, phospho-ERK1/2, total-ERK1/2, phospho-CERB and total-CREB were used. The western blots were quantifies using the Image J software after densitometric scanning of the films.

### RNA extraction and real-time PCR analysis

After 8 days of differentiation, the total RNA was extracted with QIAzol lysis reagent according to the manufacturer’s instructions. A total of 1 μg mRNA was reverse-transcribed into cDNA using High-capacity RNA-to-cDNA kit on Takara PCR Thermal Cycler (Takara, Japan). Aliquots of cDNA were amplified on a StepOnePlus Real-Time PCR system from Applied Biosystems Inc. (Marsiling Industrial Estate Road 3, Singapore) using the Power SYBR-Green PCR Master Mix from Applied Biosystems in a final volume of 20 μL. The conditions of real-time PCR were conducted as follows: denaturation at 95 °C for 10 min, 40 cycles at 95 °C for 15 s and 60 °C for 1 min. A melting curve was built in the temperature range of 60–95 °C at the end of the amplification. All primers were synthesized by Cosmo Genetech co. ltd (South Korea). The sequences of the designed primers were as follows: mPPARγ-forward-5′-GTG AAG CCC ATC GAG GAC A-3′ and reverse-5′-TGG AGC ACC TTG GCG AAC A-3′; mC/EBPα-forward-5’-GCG GGA ACG CAA CAA CAT C-3′ and reverse-5’-GTC ACT GGT CAA CTC CAG CAC-3′; mFABP4-forward-5′-AGG CTC ATA GCA CCC TCC TGT G-3′ and reverse-5’-CAG GTT CCC ACA AAG GCA TCA C-3′; mLeptin-forward-5’-GCC AGG CTG CCA GAA TTG-3′ and reverse-5’-CTG CCC CCC AGT TTG ATG-3′; mSREBP 1c-forward-5′-GGT TTT GAA CGA CAT CGA AGA-3′ and reverse-5’-CGG GAA GTC ACT GTC TTG GT-3′ and mβ-actin-forward-5′-GTG ACG TTG ACA TCC GTA AAG A-3′ and reverse-5’-GCC GGA CTC ATC GTA CTC C-3′. The mβ-actin was used as a reference gene. Target gene mRNA levels were normalized to those of β-actin using the 2^-ΔCт^ method.

### Nitro blue tetrazolium (NBT) assay

Reactive oxygen species (ROS) production on differentiated 3 T3-L1 adipocytes with or without sample treatment was measured using NBT assay. NBT assay was carried as the previously described method with some modification [24]. At Day 8 of MDI induction, 3 T3-L1 cells were incubated for 90 min in PBS containing 0.2% NBT. After washing with PBS, the stained cell plates were scanned. Thereafter, formazan, the resulted product formed by the reduction of NBT with ROS, was dissolved in DMSO, and the absorbance was measured at 520 nm using a microplate reader.

### Statistical analysis

All data are presented as the mean ± standard deviation (SD) from at least three independent experiments. Statistical significance between groups was determined using a one-way analysis of variance (ANOVA) followed by Dunnett’s multiple range tests or unpaired two-tailed Student’s t-test using GraphPad Prism 4 software. *P*-Values of < 0.05 were considered to represent statistically significant differences between groups.

## Results

### Structure identification and purity assessment of isolated 10-HDA

**Fig. 1:**
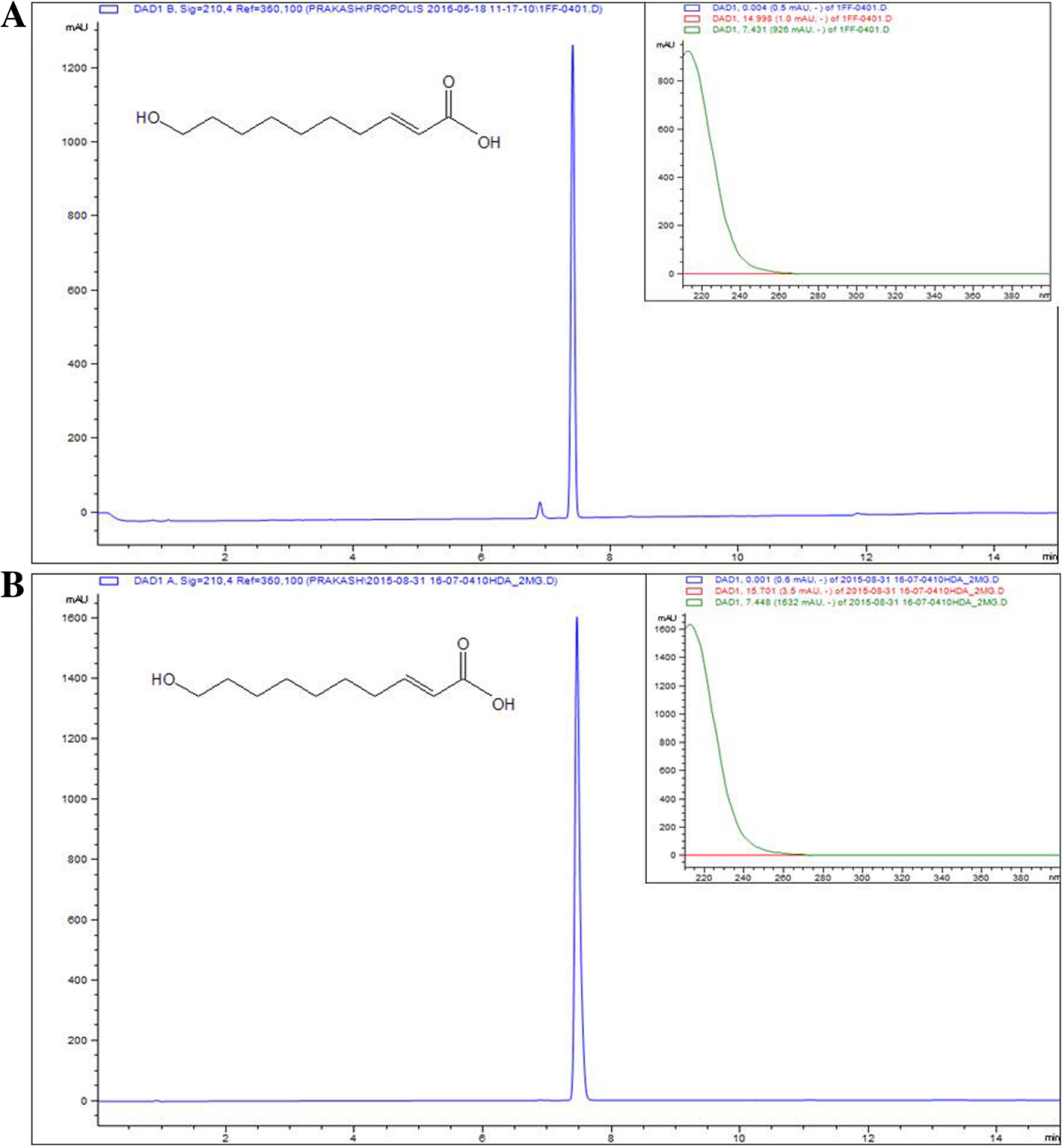
UPLC chromatogram of (**a**) isolated and (**b**) standard 10-Hydroxy-2-decenoic acid (10-HDA) with structure

The ^1^H NMR and ^13^C NMR were recorded for the identification of the isolated compound. NMR spectroscopic data showed the presence of olefinic proton. ^13^C NMR revealed the presence of 10 carbons (Additional file 1). The NMR data were compared with previously reported data [25] and the compound was confirmed to be 10-hydroxy-2*E*-decenoic acid (10-HDA). Furthermore, the mass spectrometry analysis of the isolated compound revealed an ion chromatogram ([M - H]^+^) with *m/z* 185.1149 Da, corresponding to the molecular formula C_10_H_18_O_3_ (Additional file 1). For further confirmation, the isolated compound and standard 10-HDA (Nacalai-04063-96) were injected in UPLC under similar condition. There was same retention time (7.14 min) and similar UV spectrum (*λ*max-220 nm) indicating both samples to be the same compound (Fig. 1). The purity of the isolated compound was assessed using TLC and UPLC. The compound was found to be around 95% pure. The spectroscopic data for 10-HDA are as follows: ^1^H NMR (500 MHz, DMSO-d6) ẟ ppm 6.80 (dt, *J* = 15.55, 6.97 Hz, 1 H) 5.75 (dt, *J* = 15.55, 1.39 Hz, 1 H) 3.36 (t, *J* = 6.50 Hz, 2 H) 2.12–2.20 (m, 2 H) 1.25 (m, *J* = 9.16 Hz, 10 H). ^13^C NMR (500 MHz, DMSO-*d*6): δ 175.08 (C-1), 149.44 (C-2), 167.71 (C-3), 33.07 (C-4), 29.12 (C-5), 29.25 (C-6), 29.53 (C-7), 25.98 (C-8), 31.91 (C-9), 61.28 (C-10).

### Effect of RJ, its fractions, and 10-HDA on cell viability

**Fig. 2:**
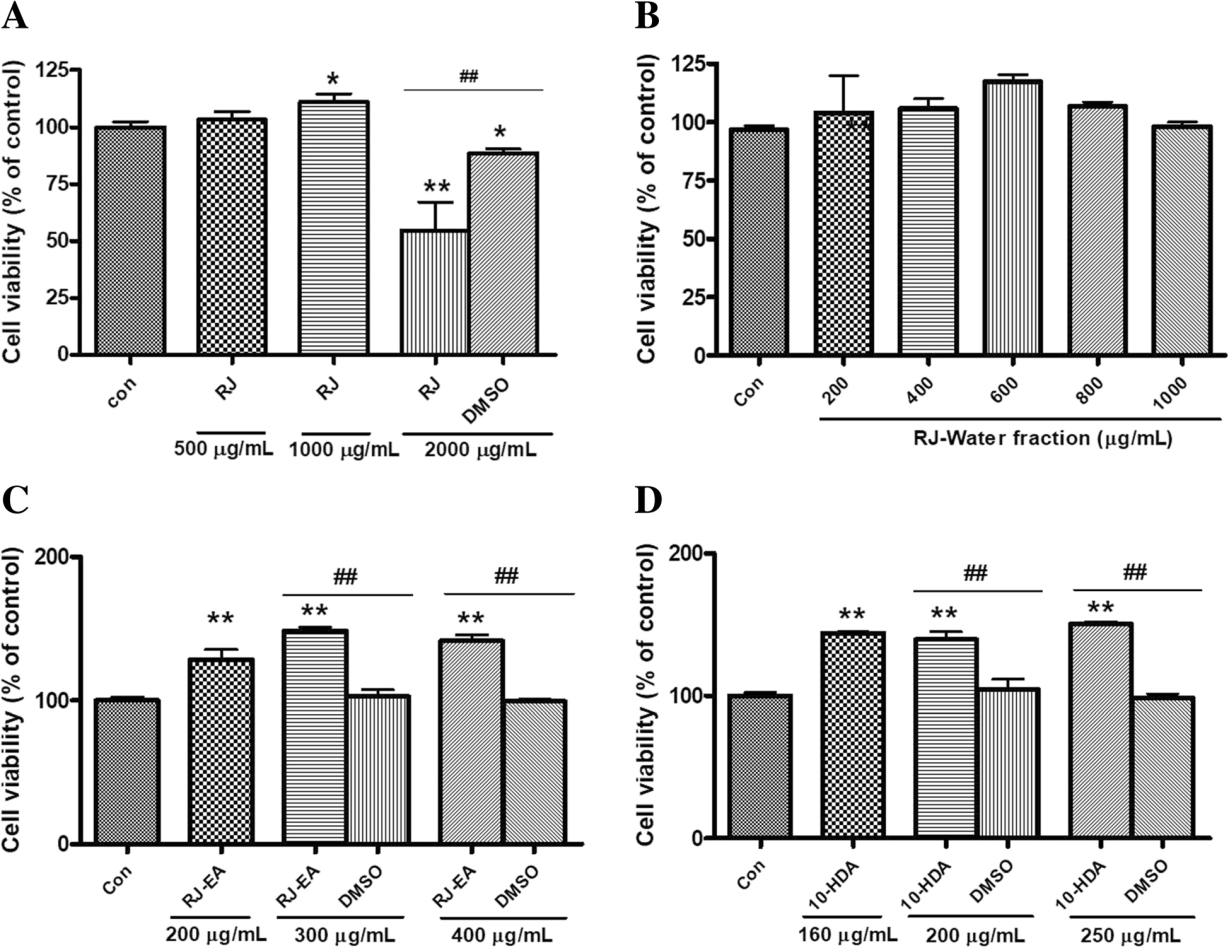
Effect of (**a**) royal jelly (RJ), (**b**) royal jelly-water fraction, (**c**) royal jelly-ethyl acetate (RJ-EA) fraction, and (**d**) 10-HDA compound on the cell viability of 3 T3-L1 preadipocytes. Data are presented as percentage viability of preadipocytes compared with untreated control. The data shown are presented as means ± SD of triplicate experiments. Statistical significance was calculated using one-way ANOVA followed by Dunnett’s multiple comparisons test. *P < 0.05 vs control; **P < 0.01 vs control. Statistical significant difference between any particular sample concentration and equivalent DMSO amount of that concentration was calculated using unpaired two-tailed Student’s t-test. ##*P* < 0.01indicates statistically significant different

Evaluation of cytotoxicity of RJ, RJ-EA fraction, RJ-water fraction, and 10-HDA on 3 T3-L1 cells was performed by MTT assay. 3 T3-L1 cells were treated with various concentrations of these samples. As shown in Fig. 2 a-d; RJ, RJ-EA fraction, RJ-water fraction, and 10-HDA were found to be non-toxic below the concentration of 1 mg/mL, 1 mg/mL, 400 μg/mL, and 250 μg/mL respectively. Moreover, the cell viability was significantly increased by RJ-EA fraction and 10-HDA in comparison with the equivalent amount of DMSO supplied to cells with respective concentrations. Thus the result suggests that RJ-EA fraction and 10-HDA aids in cell proliferation of 3 T3-L1 preadipocytes. Samples for activity assessment were prepared in the concentration within a safe range.

### Effect of RJ, RJ fractions, and 10-HDA on lipid accumulation in 3 T3-L1 adipocytes

**Fig. 3:**
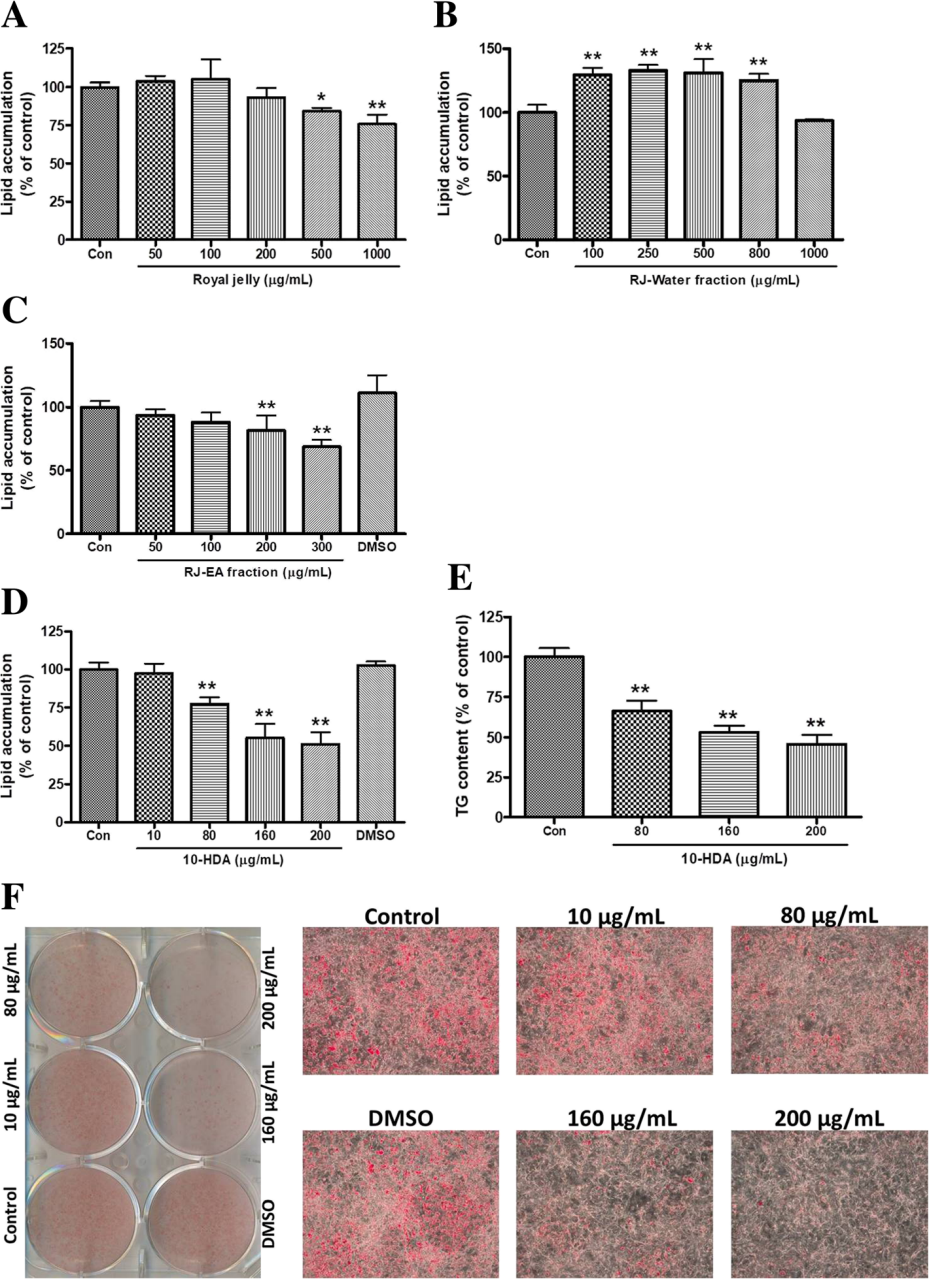
Effect of (**a**) royal jelly (RJ), (**b**) royal jelly-water fraction, (**c**) royal jelly-ethyl acetate (RJ-EA) fraction, and (**d**) 10-HDA on relative lipid accumulation by 3 T3-L1 cells. The lipid accumulations (expressed as the percentage of control) are presented as means ± SD of four separate experiments. **e** The inhibition of TG content by 10-HDA on 3 T3-L1 cells are presented as means ± SD of triplicate experiments. **f** Visualization and high magnification (× 10) were conducted for the ORO stained lipid-containing cells which were treated with 10-HDA. Statistical significance was calculated using one-way ANOVA followed by Dunnett’s multiple comparisons test. *P < 0.05 vs control; **P < 0.01vs control

The bioassay-guided isolation of anti-adipogenic compounds from RJ was initiated when RJ showed potential anti-adipogenic activity on 3 T3-L1 cell line. Lipid accumulation was assessed by ORO staining assay. RJ inhibited up to 24.2 ± 6.3% of lipid accumulation at the dose of 1 mg/mL (Fig. 3a). RJ was then fractionated into EA and water fraction and their anti-adipogenic activity on 3 T3-L1 cells were assayed. The inhibition of lipid accumulation by EA fraction was significant contrary to the no effect of water fraction (Fig. 3b and c). As EA fraction of RJ showed potential anti-adipogenic activity on 3 T3-L1 cells, a major compound on the fraction was supposed to be a root for the activity. The major compound, 10-HDA, was isolated and treated on 3 T3-L1 cells at the concentrations of 10, 80, 160 and 200 μg/mL (in DMSO) along with an equivalent amount of DMSO supplied on the highest concentration of sample (200 μg/mL). The 10-HDA showed a prominent inhibitory effect on the differentiation of 3 T3-L1 cells in a concentration-dependent manner (Fig. 3d). At Day 8 of differentiation, lipid accumulations in the cells were measured with ORO staining and stained cells were visualized and photographed using a microscope (Fig. 3f). As shown in Fig. 3d, 48.9 ± 7.9% (*p* < 0.01) of lipid accumulation was found to be reduced by 200 μg/mL of 10-HDA in comparison with untreated control. Null effect of DMSO on differentiation indicated the DMSO supplied with the drug has no role on the suppression of adipogenesis. The triacylglycerol (TG) content assay also revealed that 10-HDA suppressed the intracellular TG accumulation in differentiating 3 T3-L1 adipocytes. As shown in Fig. 3e, the highest treated dose of 10-HDA suppressed intracellular TG content by 54.4 ± 6.0% (*p* < 0.01) compared to untreated control.

### Effect of 10-HDA on protein expression of specific adipogenic transcription factors during adipogenesis in 3 T3-L1 adipocytes

**Fig. 4:**
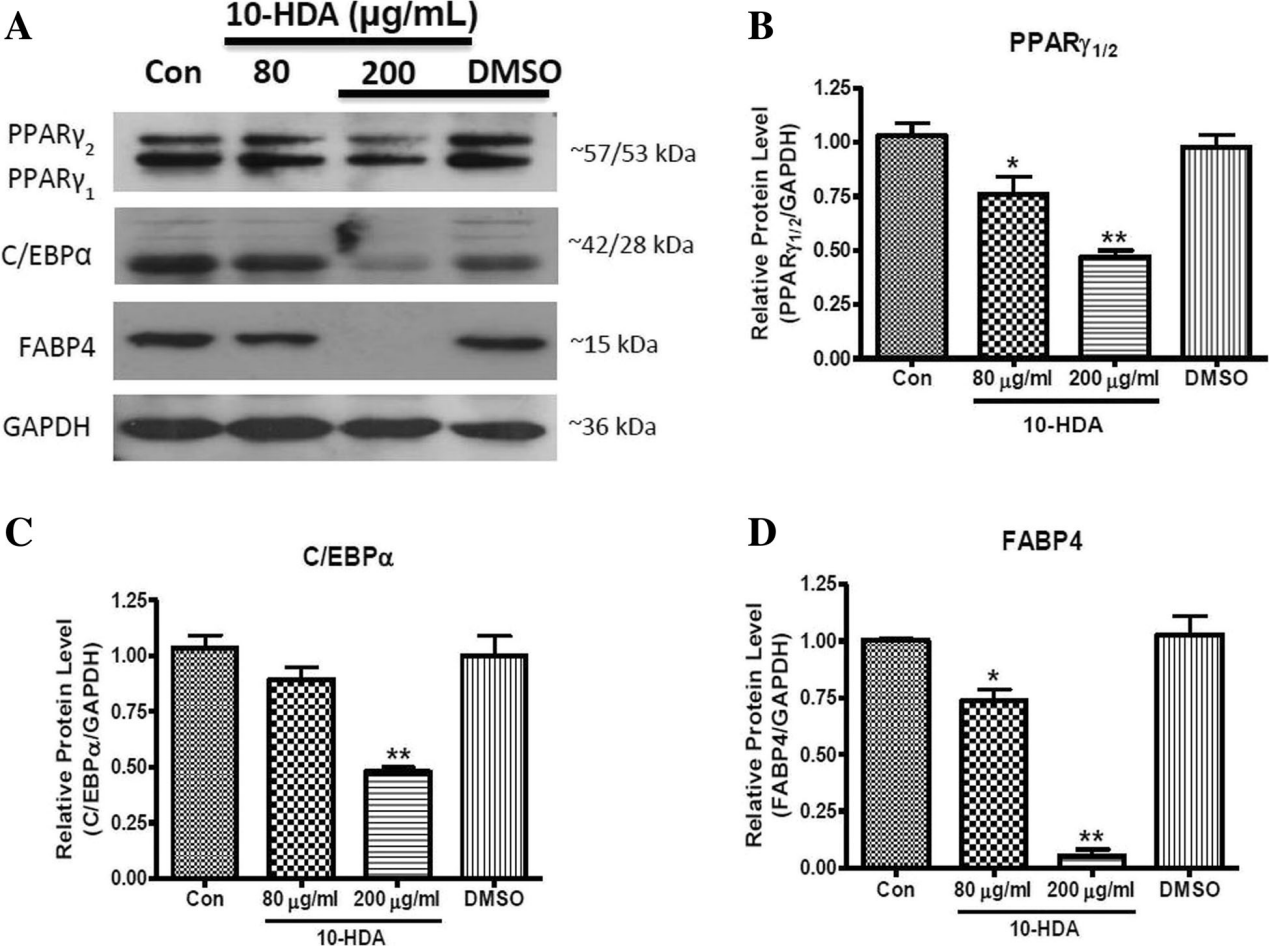
Effects of 10-HDA on protein expression level of PPARγ, C/EBPα, and FABP4 during 3 T3-L1 adipocyte differentiation. **a** The blot represented the protein expression levels of different transcription factors. The band intensities of (**b**) PPARγ, (**c**) C/EBPα, and (**d**) FABP4 were measured and normalized to GAPDH. The data shown are presented as means ± SD of triplicate experiments. Statistical significance was calculated using one-way ANOVA followed by Dunnett’s multiple comparisons test. *P < 0.05 vs control; **P < 0.01 vs control

Adipocyte differentiation requires sequential activation of multiple pro-adipogenic transcription factors including PPARγ, C/EBPα/β, and FABP4 [26]. Thus, the protein levels were examined to identify whether the reduced fat accumulation in adipocytes by 10-HDA was due to the down-regulation of the aforementioned adipogenic transcription factors. Different concentrations of 10-HDA (80 and 200 μg/mL) were treated at Day 0 and cell lysates were harvested at Day 8 for western blot analysis. Cells on a separate well were treated with plain DMSO in an equivalent amount that was supplied in the treatment of highest concentration (200 μg/mL) of 10-HDA to determine whether the effect was due to DMSO. Result showed that the 10-HDA significantly down-regulated the protein expression of PPARγ (Fig. 4a and b), C/EBPα (Fig. 4a and c), and FABP4 (Fig. 4a and d) in a concentration-dependent manner compared to the control, suggesting that the 10-HDA inhibits adipogenesis by down-regulation of PPARγ, C/EBPα, and FABP4 expression.

### Effect of 10-HDA on gene expression of specific adipogenic transcription factors during adipogenesis in 3 T3-L1 adipocytes

**Fig. 5:**
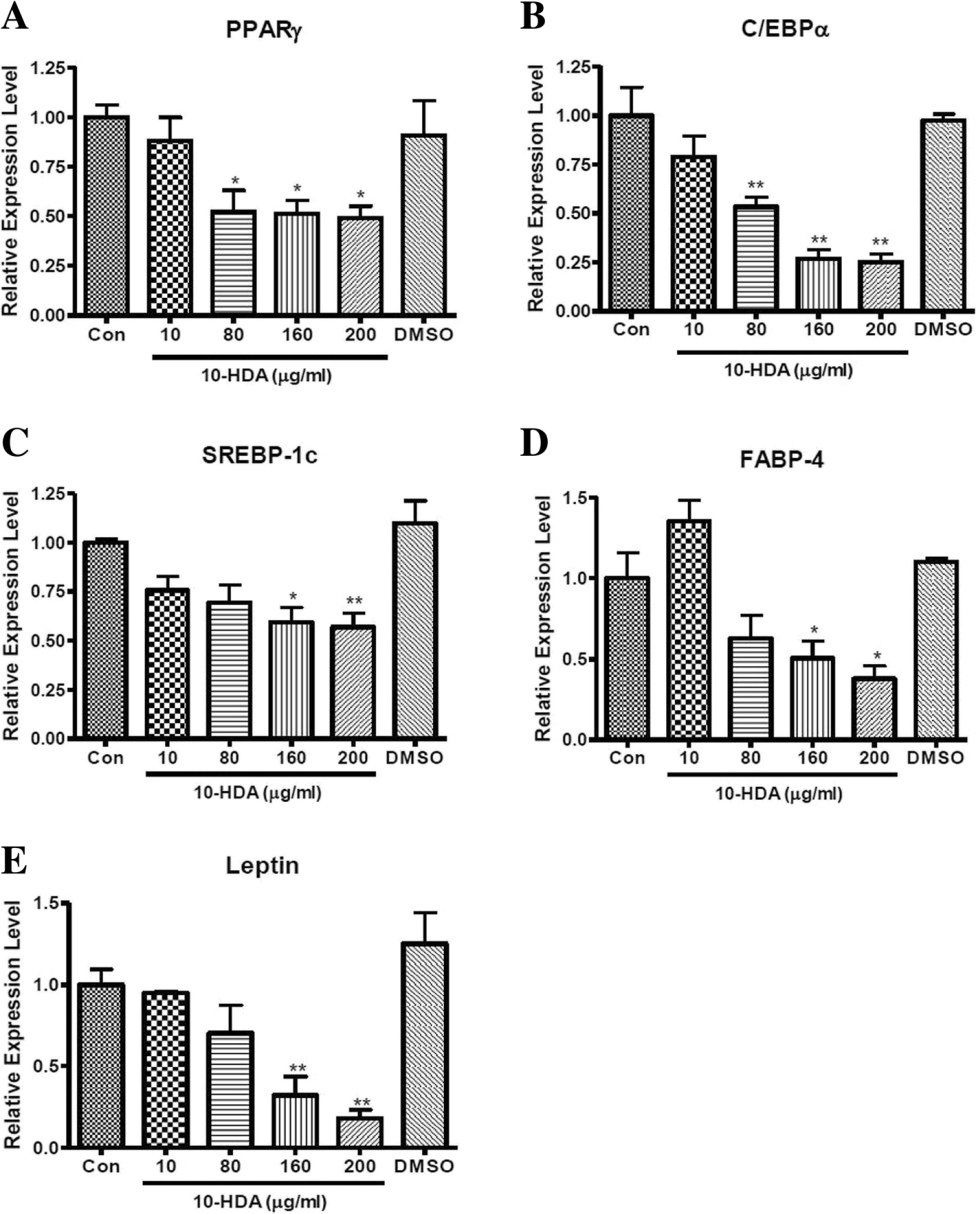
Effects of 10-HDA on gene expression level of (**a**) PPARγ, (**b**) C/EBPα, (**c**) SREBP-1c, (**d**) FABP4, and (**e**) Leptin during 3 T3-L1 adipocyte differentiation. Each gene expression levels were quantified and normalized to β-actin. The data shown are presented as means ± SEM of triplicate experiments. Statistical significance was calculated using one-way ANOVA followed by Dunnett’s multiple comparisons test. *P < 0.05 vs control; **P < 0.01 vs control

As the protein expression level of key adipogenic transcription factors was found to be significantly down-regulated by 10-HDA (Fig. 4), the effect of 10-HDA on gene expression level of these transcription factors was further examined by real-time PCR. The 3 T3-L1 adipocytes were treated with 10, 80, 160 and 200 μg/mL concentrations of 10-HDA and total mRNA was extracted at Day 8 of the sample treatment. On a separate well, cells were treated with plain DMSO in an equivalent amount that was used in the treatment of highest concentration (200 μg/mL) of 10-HDA to see whether the effect was due to DMSO. The mRNA expression of PPARγ, C/EBPα, SREBP-1c, FABP4, and Leptin were determined. As shown in Fig. 5, the gene expression of the aforementioned transcription factors were markedly decreased by 10-HDA in a concentration-dependent manner compared to the control.

### Effect of 10-HDA on an early stage of adipogenesis in 3 T3-L1 adipocytes

**Fig. 6:**
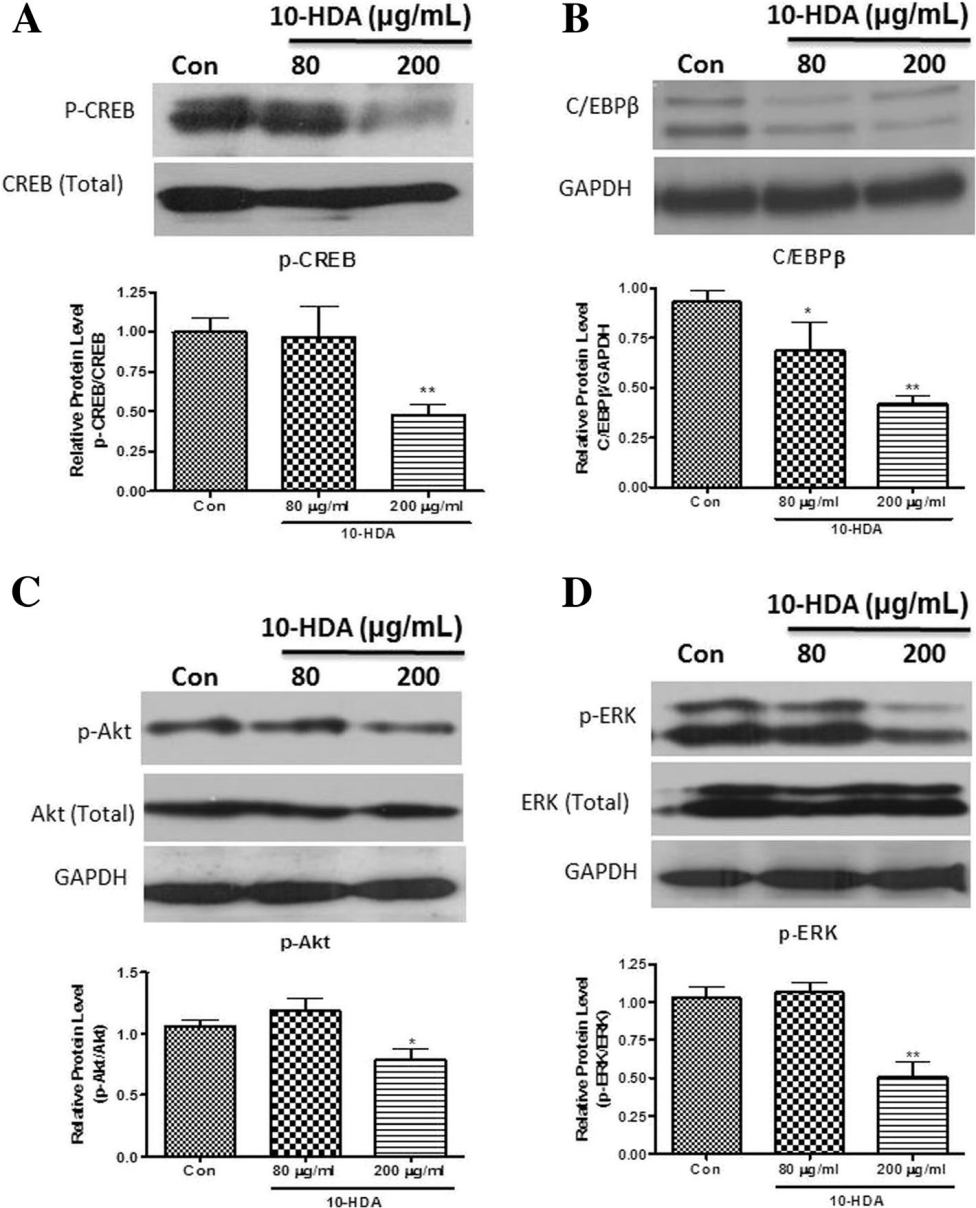
Effect of 10-HDA on the early stage of adipogenesis in 3 T3-L1 adipocytes. The blot represented the protein expression levels of (**a**) p-CREB, (**b**) C/EBPβ, (**c**) p-Akt, and (**d**) p-ERK. The band intensities of (**a**) p-CREB, (**c**) p-Akt, and (**d**) p-ERK were measured and normalized to total protein level of the respective protein, whereas, the band intensity of (**b**) C/EBPβ was normalized to GAPDH. The data shown are presented as means ± SD of triplicate experiments. Statistical significance was calculated using one-way ANOVA followed by Dunnett’s multiple comparisons test. *P < 0.05 vs control; **P < 0.01 vs control

As the protein and gene expression level of key adipogenic transcription factors was found to be significantly down-regulated by 10-HDA (Figs. 4 and 5), the effect of 10-HDA on preliminary molecules such as phospho-ERK, phospho-Akt, phospho-CERB, and C/EBPβ was studied at the early stage of adipogenesis to see whether the down-regulation of the key transcription factors by 10-HDA was due to down-regulation of the aforementioned preliminary molecules. The protein expression of p-CREB and C/EBPβ was evaluated in 3 T3-L1 cells by immunoblotting assay after treatment with 80 and 200 μg/mL of 10-HDA for 36 h. As shown in Fig. 6, the expressions of p-CREB (Fig. 6a), and C/EBPβ (Fig. 6b) were markedly down-regulated with 10-HDA.

In a previous study p-ERK1/2 and p-Akt expression in 3 T3-L1 cells was found to be maximized at around 30 min and 2 h of MDI induction respectively [17]. Thus, the protein expression level of p-ERK and p-Akt was examined in the preadipocytes harvested respectively at 30 min and 2 h of the drug treatment. The result showed the 10-HDA had a significant effect in down-regulation of both p-Akt (Fig. 6c) and p-ERK1/2 (Fig. 6d) in 3 T3-L1 preadipocytes.

### Effect of 10-HDA on ROS production during 3 T3-L1 adipocyte differentiation

**Fig. 7:**
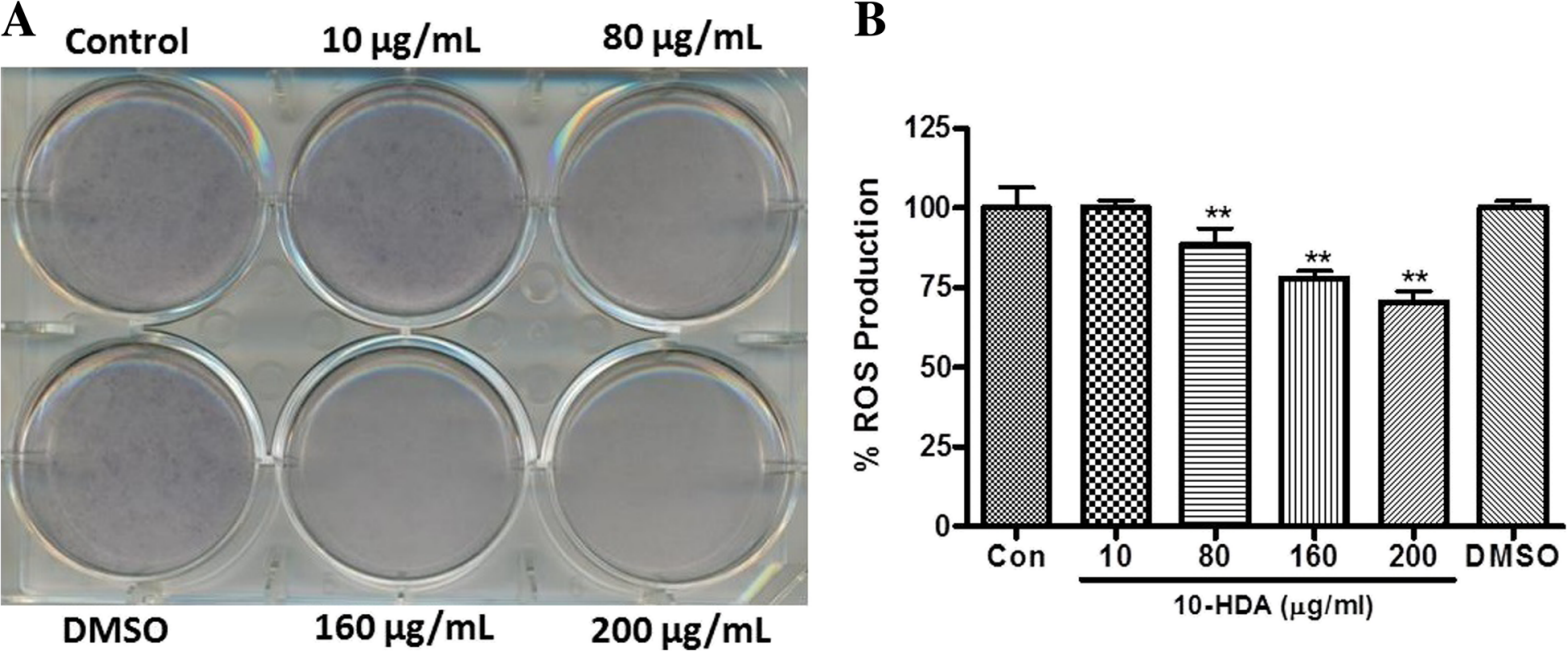
Effect of 10-HDA on ROS production during 3 T3-L1 adipocyte differentiation. **a** Visualization was conducted for the NBT stained cells which were treated with 10-HDA. **b** The ROS production was quantified as percent of control. The ROS productions are presented as means ± SD of triplicate experiments. Statistical significance was calculated using one-way ANOVA followed by Dunnett’s multiple comparisons test. **P < 0.01vs control

Previous studies demonstrated that fat accumulation in adipocytes is correlated with systemic oxidative stress in obesity [27]. Therefore, the effect of 10-HDA on reactive oxygen species (ROS) production in fully differentiated 3 T3-L1 cells was estimated by NBT assay. NBT can be reduced by ROS to formazan, which is dark blue and insoluble [24]. The production of formazan, representing ROS production, was markedly suppressed in concentration-dependent manner by 10-HDA at Day 8 of MDI induction in 3 T3-L1 adipocytes (Fig. 7 a and b). Around 29.6 ± 3.3% (*p* < 0.01) decrease in ROS production was seen in 200 μg/mL of 10-HDA in comparison with untreated control.

## Discussion

Obesity is a major health and economic problem worldwide. It is characterized by multiple metabolic complications like type 2 diabetes. Most of the anti-obesity drugs have serious cardiac as well as psychological adverse effects [14]. Therefore, it is essential to develop a natural therapy for obesity with minimal adverse effects. Royal Jelly (RJ) is increasingly used as a dietary supplement due to its multiple biological benefits [4, 28]. It is widely used both in folk and in authorized medications [5]. Use of RJ as anti-obesity medicine has not been reported yet. In this study, we evaluated the anti-obesity potential of RJ. The result showed significant inhibition of lipid accumulation on 3 T3-L1 adipocyte by RJ. Further study on RJ led to the isolation of a major compound having potential anti-adipogenic activity. The active compound was identified as 10-HDA from the spectroscopic and chromatographic analysis.

Adipogenesis is a complex process that requires the synergistic action of various transcription factors and adipogenic markers including PPARγ, C/EBPα, SREBP-1c, and FABP4. The expression of PPARγ and C/EBPα cross-regulate each other through positive feedback. That synergy induces differentiation markers, such as FABP4, which are associated with binding of free fatty acids (FFA) and triacylglycerol (TG) and that leads to the appearance of lipid droplets into adipocytes [26, 29]. Moreover, SREBP-1c, an SREBP isoform, also activates PPARγ by inducing its expression [30]. Thus the adipocytes are maintained in terminal differentiation stage by PPARγ [31]. In the current study, 10-HDA showed significant down-regulation of the aforementioned transcription factors and adipogenic markers (Figs. 4 and 5). Leptin is one of the adipokines secreted mainly by adipocytes. Its secretion directly depends on the mass of adipose-tissue, TG content, and the nutritional environment to adipocytes [32]. Leptin levels are considered to increase with adipogenesis in 3 T3-L1 adipocytes [33]. In this study, 10-HDA significantly decreased leptin gene expression levels in 3 T3-L1 adipocytes (Fig. 5e), indicating its potential anti-adipogenic activity.

**Fig. 8:**
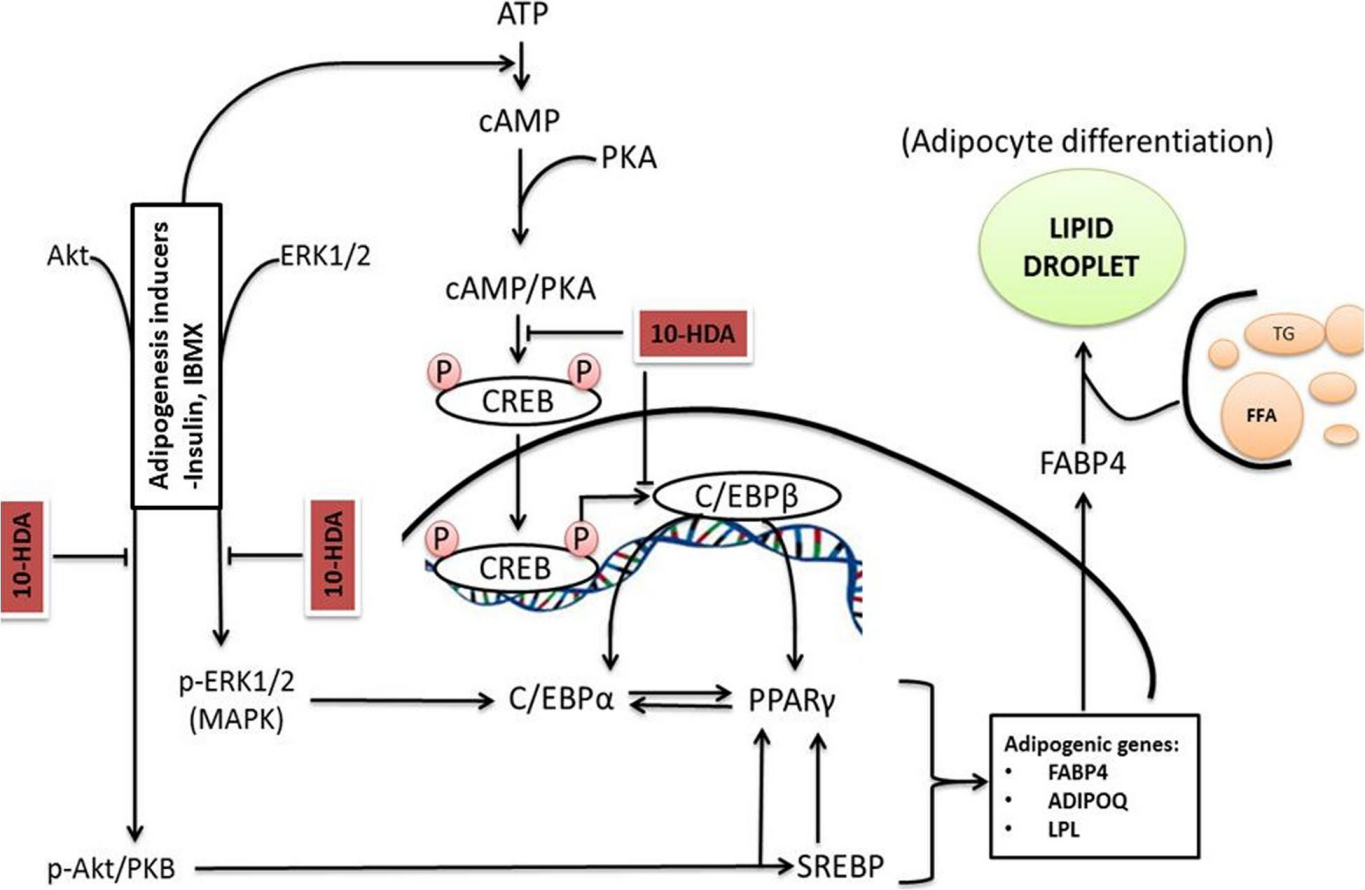
Schematic diagram on the molecular mechanism of the anti-adipogenic effect of 10-HDA in 3 T3-L1 preadipocytes. 10-HDA inhibited the accumulation of lipid droplets within 3 T3-L1 adipocyte via two possible mechanisms; by inhibiting cAMP/PKA pathway and p-Akt and MAPK dependent insulin signaling pathway. ATP: adenosine triphosphate; PKA: protein kinase A; MAPK: mitogen-activated protein kinase; ADIPOQ: adiponectin precursor gene; LPL: lipoprotein lipase; FFA: free fatty acids; TG: triacylglycerol

cAMP/PKA pathway plays a fundamental role during the early stages of adipogenesis [34]. Cyclic adenosine monophosphate (cAMP) are elevated by differentiation-inducing agents such as insulin and IBMX in preadipocytes [35]. cAMP now in association with protein kinase A (PKA) stimulates phosphorylation of cAMP response element binding protein (CREB), thus increasing its transcriptional activity. Such activated CREB further up-regulates the expression of C/EBPβ [16]. C/EBPβ binds at cis-C/EBP regulatory element which is exposed by both the C/EBPα and PPARγ genes in their proximal promoter site and ultimately activates their transcription [36, 37]. The PPARγ and C/EBPα principally regulate terminal differentiation of adipocytes [35]. Furthermore, it is also reported that CREB directly promotes PPARγ2 gene transcription [36]. In this study, 3 T3-L1 preadipocytes were treated with 10-HDA to evaluate its effect on the early stages of differentiation. Our results showed that 10-HDA inhibit the initiation of adipogenesis by down-regulating protein expression level of the CREB and C/ EBPβ (Fig. 6a and b). Therefore, it suggests that 10-HDA inhibits adipogenesis via cAMP/PKA pathway (Fig. 8).

During adipogenesis, PPARγ is targeted by many adipogenic signaling pathways [35]. A p-Akt and MAPK dependent insulin signaling pathway is considered one of the key cascades for adipogenesis in adipocytes. Insulin independently induces phosphorylation of Akt and ERK1/2 [38]. Phosphorylation of Akt leads to activation of SREBP-1c [39]. SREBP-1c further regulates activation of PPARγ [40]. In addition, Akt activation enhances expression of PPARγ by reducing the expression of forkhead transcriptional factor 1 (Foxo1), an antagonist of PPARγ [41]. Moreover, Akt phosphorylation activates C/EBPα during preadipocyte differentiation [42]. Additionally, it was reported that C/EBPα and PPARγ in 3 T3-L1 adipocytes was reduced by ERK antagonists. In addition, insulin in MDI activates ERK1/2 [43]. This evidence showed that insulin-induced ERK1/2 has an important role in the activation of transcription factors, C/EBPα and PPARγ. Thus, the down-regulation of key transcription factors in 3 T3-L1 adipocytes was possible because of the inhibition of Akt and ERK1/2 phosphorylation by 10-HDA in the earliest phase of differentiation (Fig. 6c and d). Therefore, by correlating our results with previous scientific pieces of evidence, we found that the anti-adipogenic activity of 10-HDA mediated via two mechanisms: inhibition of cAMP/PKA pathway and inhibition of p-Akt and MAPK dependent insulin signaling pathway (Fig. 8).

An imbalance between the production and elimination of reactive species (ROS) in body cause oxidative stress. An accumulation of ROS in adipose tissue is one of the initial events in the occurrence of metabolic syndrome in obesity [24]. ROS may be involved in both initiations of pre-adipocyte differentiation and maturation of adipocytes in terminal phase [35]. Oxidation of the accumulated lipid molecules in adipocytes is one of the major sources of ROS production in adipocytes [44]. Increased oxidative stress such produced is a critical pathogenic mechanism of obesity-associated metabolic complications [24]. Since 10-HDA showed significant inhibition of ROS production in 3 T3-L1 adipocytes (Fig. 7), it can be considered to have beneficial effects of the 10-HDA against obesity-linked oxidative stress.

## Conclusion

From overall results, the main anti-adipogenic component in RJ was found to be 10-HDA. We also proposed possible mechanisms for the anti-adipogenic activity of 10-HDA, which involved the inhibition of cAMP/PKA pathway, and p-Akt and MAPK dependent insulin signaling pathway. Hence, 10-HDA can be a potential medication for the treatment of obesity. In addition, RJ being an abundant source of 10-HDA has great potential to be an excellent natural functional food for obese people.

## Additional file

Additional file 1: Data on identification and purity assessment of isolated 10-Hydroxy-2-decenoic acid (10-HDA). **Figure S1.** 1H NMR Spectrum (A) and 13C NMR (500 MHz) spectra (B) of the compound isolated from ethyl acetate fraction of RJ in DMSO-d6; **Figure S2.** TLC analysis of Royal jelly (RJ), RJ-EA fraction (EA), isolated 10-HDA compound (Com) and standard 10-HDA (Std); **Figure S3.** Mass spectrometry - structure of the 10-Hydroxy-2-decenoic acid (10-HDA). (DOCX 156 kb)

## Abbreviations

10-HDA: 10-hydroxy-2*E*-decenoic acid
^13^C NMR: Carbon nuclear magnetic resonance
^1^H-NMR: Proton nuclear magnetic resonance
C/EBPα: CAAT/enhancer binding protein alpha
C/EBPβ: CAAT/enhancer binding protein beta
cAMP: Cyclic adenosine monophosphate
CREB: cAMP response element binding protein
DMEM: Dulbecco's Modified Eagle Medium
DMSO: Dimethyl sulfoxide
EA: Ethyl acetate
FABP4: Adipocyte-specific fatty acid binding proteins
FBS: Foetal bovine serum
FFA: Free fatty acids
GAPDH: Glyceraldehyde-3-phosphate dehydrogenase
IBMX: 3-Isobutyl-1-methylxanthine
MAPK: Mitogen-activated protein kinase
MDI: Methylisobutylxanthine, dexamethasone, insulin
MTT: Thiazolyl blue tetrazolium bromide
NBT: Nitro blue tetrazolium
NCS: Newborn calf serum
ORO: Oil-red O
p-Akt: Phosphorylated-Akt
p-ERK: Phosphorylated-ERK1/2
PPARγ: Peroxisome proliferator-activated receptor gamma
RJ: Royal jelly
ROS: Reaction oxygen species
SREBP-1c: Sterol response element binding protein- 1c
TG: Triacylglycerol
TLC: Thin layer chromatography
UPLC: Ultra-performance liquid chromatography
WAT: White adipose tissue
