# Supplementary material for: Bioassay-guided isolation of active anti-adipogenic compound from royal jelly and the study of possible mechanisms
Source: BMC Complement Altern Med. 2019 Jan 29;19:33. doi: 10.1186/s12906-018-2423-2 (PMC6352437; doi:10.1186/s12906-018-2423-2)
Supplement: Supplementary file 1 — Data on identification and purity assessment of isolated 10-Hydroxy-2-decenoic acid (10-HDA). Figure S1. 1H NMR Spectrum (A) and 13C NMR (500 MHz) spectra (B) of the compound isolated from ethyl acetate fraction of RJ in DMSO-d6; Figure S2. TLC analysis of Royal jelly (RJ), RJ-EA fraction (EA), isolated 10-HDA compound (Com) and standard 10-HDA (Std); Figure S3. Mass spectrometry - structure of the 10-Hydroxy-2-decenoic acid (10-HDA). (DOCX 156 kb) [file 12906_2018_2423_MOESM1_ESM.docx]

**Additional file 1**

**Supplementary data description**

## Identification and purity assessment of isolated 10-Hydroxy-2-decenoic acid

10-Hydroxy-2-decenoic acid (10-HDA) was obtained as white colored crystals. The ^1^H NMR and ^13^C NMR were recorded for identification of the compound (Fig. 1A-B). Further identification and purity assessment was done by comparing chromatograms of the isolated compound and standard 10-hydroxy-2*E*-decenoic acid (Nacalai, Japan) using TLC and UPLC. In TLC the R*f*-value was found to be similar for both isolated and standard compounds (Fig. 2A-3C). Furthermore, retention time of both isolated and standard compounds peak on UPLC was also found to be similar. Mass spectrometry analysis of the active isolated compound revealed an ion chromatogram ([M - H]^+^) with *m/z* 185.1149 Da, corresponding to the molecular formula C_10_H_18_O_3_ (Fig. 3). Analyzing all data, it was confirmed the isolated compound was 10-Hydroxy-2-decenoic acid (10-HDA). Purity of the isolated compound was assessed using TLC and UPLC. The compound was found to be 94.97% pure.

10-Hydroxy-2-decenoic acid: ^1^H NMR (500 MHz, DMSO-d6) ẟ ppm 6.80 (dt, *J*=15.55, 6.97 Hz, 1 H) 5.75 (dt, *J*=15.55, 1.39 Hz, 1 H) 3.36 (t, *J*=6.50 Hz, 2 H) 2.12 - 2.20 (m, 2 H) 1.25 (m, *J*=9.16 Hz, 10 H). ^13^C NMR (500 MHz, DMSO-*d*6): δ 175.08 (C-1), 149.44 (C-2), 167.71 (C-3), 33.07 (C-4), 29.12 (C-5), 29.25 (C-6), 29.53 (C-7), 25.98 (C-8), 31.91 (C-9), 61.28 (C-10).

**Supplementary Figures**


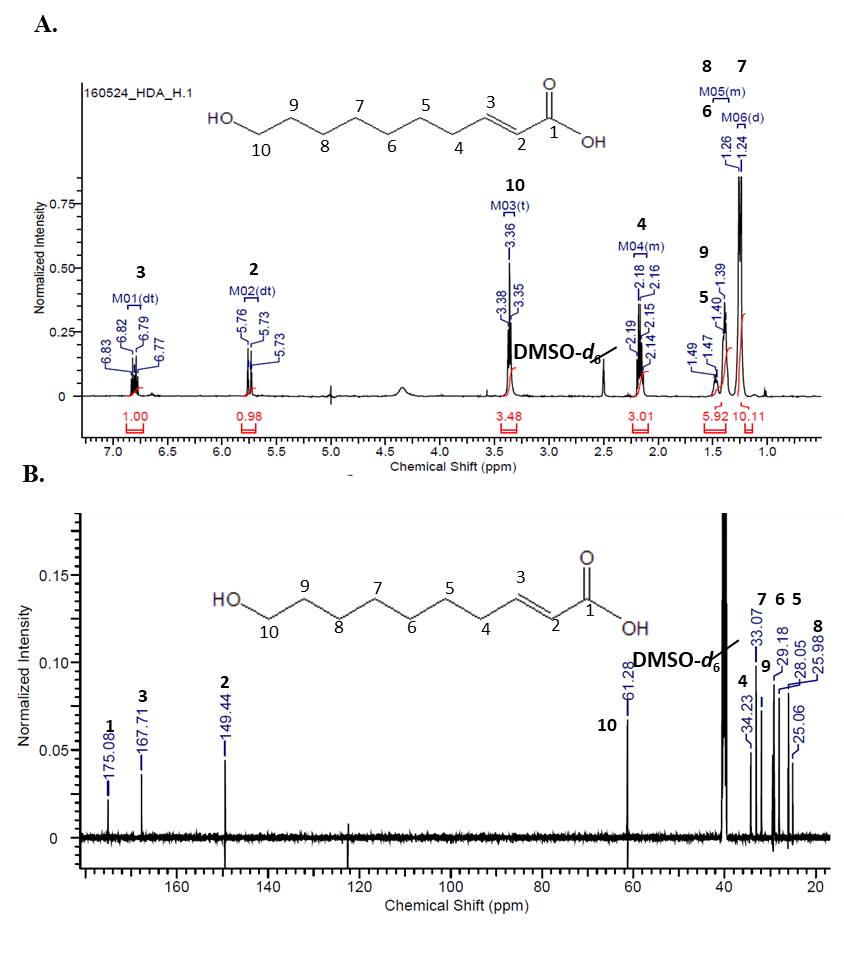


Figure S1 ^1^H NMR Spectrum (A) and ^13^C NMR (500 MHz) spectra (B) of the compound isolated from ethyl acetate fraction of RJ in DMSO-d6.


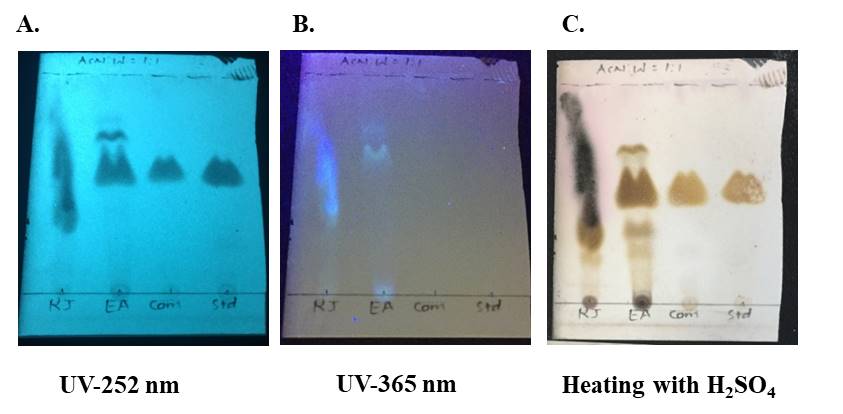


Figure S2 TLC analysis of Royal jelly (RJ), RJ-EA fraction (EA), isolated 10-HDA compound (Com) and standard 10-HDA (Std). TLC was eluted with the solvent mixture, acetonitrile: water =1: 1, v/v.

**
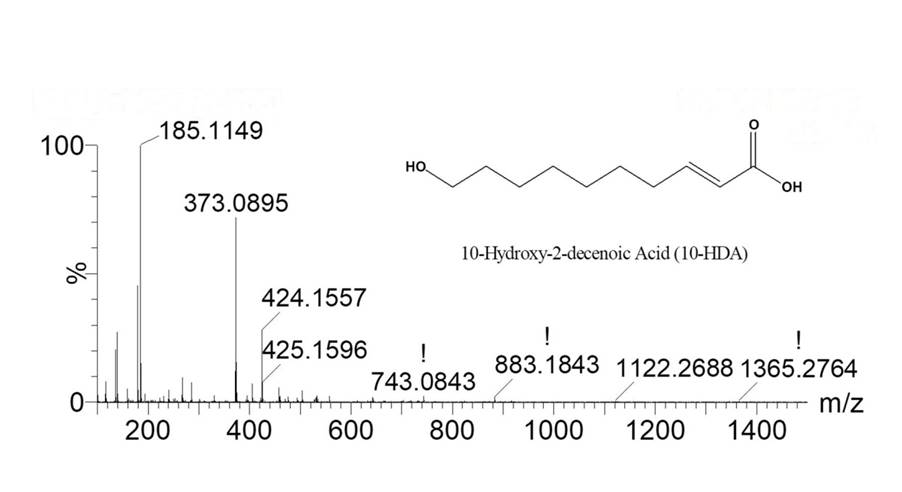
**

Figure S3 Mass spectrometry - structure of the 10-Hydroxy-2-decenoic acid (10-HDA)
